# Tapered whisker reservoir computing for real-time terrain identification-based navigation

**DOI:** 10.1038/s41598-023-31994-x

**Published:** 2023-03-30

**Authors:** Zhenhua Yu, S. M. Hadi Sadati, Shehara Perera, Helmut Hauser, Peter R. N. Childs, Thrishantha Nanayakkara

**Affiliations:** 1 Dyson School of Design Engineering, Imperial College London, London, SW7 2DB UK; 2 Department of Surgical and Interventional Engineering, King’s College London, London, WC2R 2LS UK; 3 Bristol Robotics Laboratory, and also with SoftLab, University of Bristol, Bristol, BS8 1TH UK

**Keywords:** Mechanical engineering, Computational science

## Abstract

This paper proposes a new method for real-time terrain recognition-based navigation for mobile robots. Mobile robots performing tasks in unstructured environments need to adapt their trajectories in real-time to achieve safe and efficient navigation in complex terrains. However, current methods largely depend on visual and IMU (inertial measurement units) that demand high computational resources for real-time applications. In this paper, a real-time terrain identification-based navigation method is proposed using an on-board tapered whisker-based reservoir computing system. The nonlinear dynamic response of the tapered whisker was investigated in various analytical and Finite Element Analysis frameworks to demonstrate its reservoir computing capabilities. Numerical simulations and experiments were cross-checked with each other to verify that whisker sensors can separate different frequency signals directly in the time domain and demonstrate the computational superiority of the proposed system, and that different whisker axis locations and motion velocities provide variable dynamical response information. Terrain surface-following experiments demonstrated that our system could accurately identify changes in the terrain in real-time and adjust its trajectory to stay on specific terrain.

## Introduction

Terrain identification is an important fundamental function for autonomous mobile robots’ navigation and performing tasks such as auto-patrolling, automatic driving, and back-country rescue in extreme, unstructured environments. By real-time identifying of different terrain surfaces and perceiving terrain texture information, mobile robots can dynamically adjust their initial planning trajectory for safer and more efficient navigation. For that, a robot needs to be able to actively perceive the terrain accurately and quickly, with low computing cost in order to support real-time applications^1^.

To achieve accurate recognition of terrain, convolutional neural network-based approaches have been widely proposed and investigated by using information from a single type of sensor or sensor fusion. Based on different sensing modalities, these methods can be classified into two main groups: exteroceptive-based^2–4^ and proprioceptive-based^5,6^. The most popular exteroceptive method is vision-based^7^, which enables robots to recognize different terrains in advance at longer distances to avoid unwanted consequences such as sinking into the soft sand. Pedro et al.^8^ proposed a stereo-based method to detect various sizes of obstacles for all-terrain environments exploiting their complementary properties with high detection accuracy. Other attempts have been made to address terrain classification using LIDAR data to help mobile robots operate safely in challenging off-road environments^9,10^. Zhou et al.^11^ presented a self-supervised approach by employing both LIDAR and vision sensing to identify terrain surfaces for robot autonomous navigation in a forest. Even though the above methods have achieved quite good results, their accuracy can be affected by the visual appearance of the terrains, due to changes in light intensity at different times, different visibility conditions (e.g., snow, smoke, fog), or different coverings (e.g., water, dirt, fallen leaves, snow) of the terrain.

To compensate for the limitations of vision-based terrain classification, various methods have been proposed to achieve high-accuracy terrain classification eliminating the effects of the terrain’s visual appearance by using proprioceptive information generated by the mobile robot’s dynamical contact with the terrain, such as the vibration^12,13^, wheel-terrain audio^5,14^, and tactile signals^15,16^. These methods extract vibration feature signals to train neural networks for terrain classification by: (1). extracting time domain features directly in the time domain^15^, (2). extracting frequency domain features^17^ or power spectrum by Fourier transform^14^, (3). other statistical methods^12,16^, which have reported good performance in several environments. However, the proprioceptive features collected for terrain classification are sometimes susceptible to robot body self-vibration, which often affects the robot’s perceptual accuracy.

**Figure 1:**
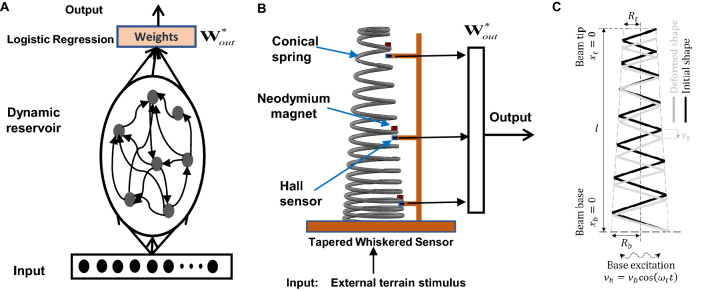
(**A**) Schematic diagram of the traditional (physical)reservoir computing system (**B**) Our proposed tapered whisker-based reservoir computing system. The mechanical spring is exploited as a reservoir. The input is coming from the interaction of the robot with different terrains. The readouts are implemented as three Hall sensors located at different locations. The learning consists of finding three corresponding linear output weights $W_{{\textrm{out}}}$, which significantly reduces the required computation. (**C**) The diagram for the axial vibration of a tapered beam under base harmonic excitation when the mobile robots move on different surfaces. $\nu$ denotes the spring local axial displacement.

While these ongoing research areas have demonstrated that each modality is effective in identifying different terrain types, these methods mentioned above still suffer from classification and ambiguity. Therefore, terrain classification methods based on multi-sensor information fusion have been investigated. These methods can be divided into two main categories: exteroceptive-exteroceptive sensor fusion^18,19^ and exteroceptive-proprioceptive sensor fusion^20–22^. Milella et al.^18^ presented a self-learning framework that employs a radar classifier to assign training labels for the visual-based classification module, but its performance may still be influenced by light levels. Typically, terrain classification by combining exteroceptive modalities (e.g. images, lidar, radar) and proprioceptive modalities (e.g. acceleration, audio) could have better environmental robustness and achieve better performance. For example, a self-supervised terrain classification framework by combining the visual and audio modalities was proposed^20^, which used the audio feature to self-label the visual terrain images for semantic segmentation.

Another interesting branch of the proprioceptive sensor is to use artificial whiskers for mobile robot navigation^23^, object detection^24^, obstacle shape recognition^25^, and terrain surface information^26^ inspired by animals using their whiskers to sense environmental information and navigate in the dark environments^27^. Zurek et al.^28^ showed that a mobile robot could utilize static antennae as a local detector to determine the orientation and location of sudden obstacles during quick motion, which could compensate for the limitation of visual methods. Moreover, Solomon et al.^29^ proposed an approach to perceive the object’s 3D shape information by using a tactile whisker to collect contact torque information for obstacle shape recognition. These results demonstrate the potential that a whisker sensor could be developed for terrain perception in an extreme environment.

However, most of the aforementioned state-of-the-art algorithms require a large number of training data, which is typically difficult to obtain. Moreover, it involves manual labelling to build a training dataset, which is not trivial in complex, unknown environments. Furthermore, these methods need a significant amount of computational resources for data processing e.g, Fourier transformation and online neural network training, and their classification performance tends to deteriorate when there is no sufficient variability in the training dataset^30^. As a result, quickly and cost-effectively identifying and predicting the surface characteristics of an unknown terrain in complex extreme environments such as Mars is difficult for practical robotic applications, especially, with IMU and vision methods which require complicated processing of raw data.

**Figure 2:**
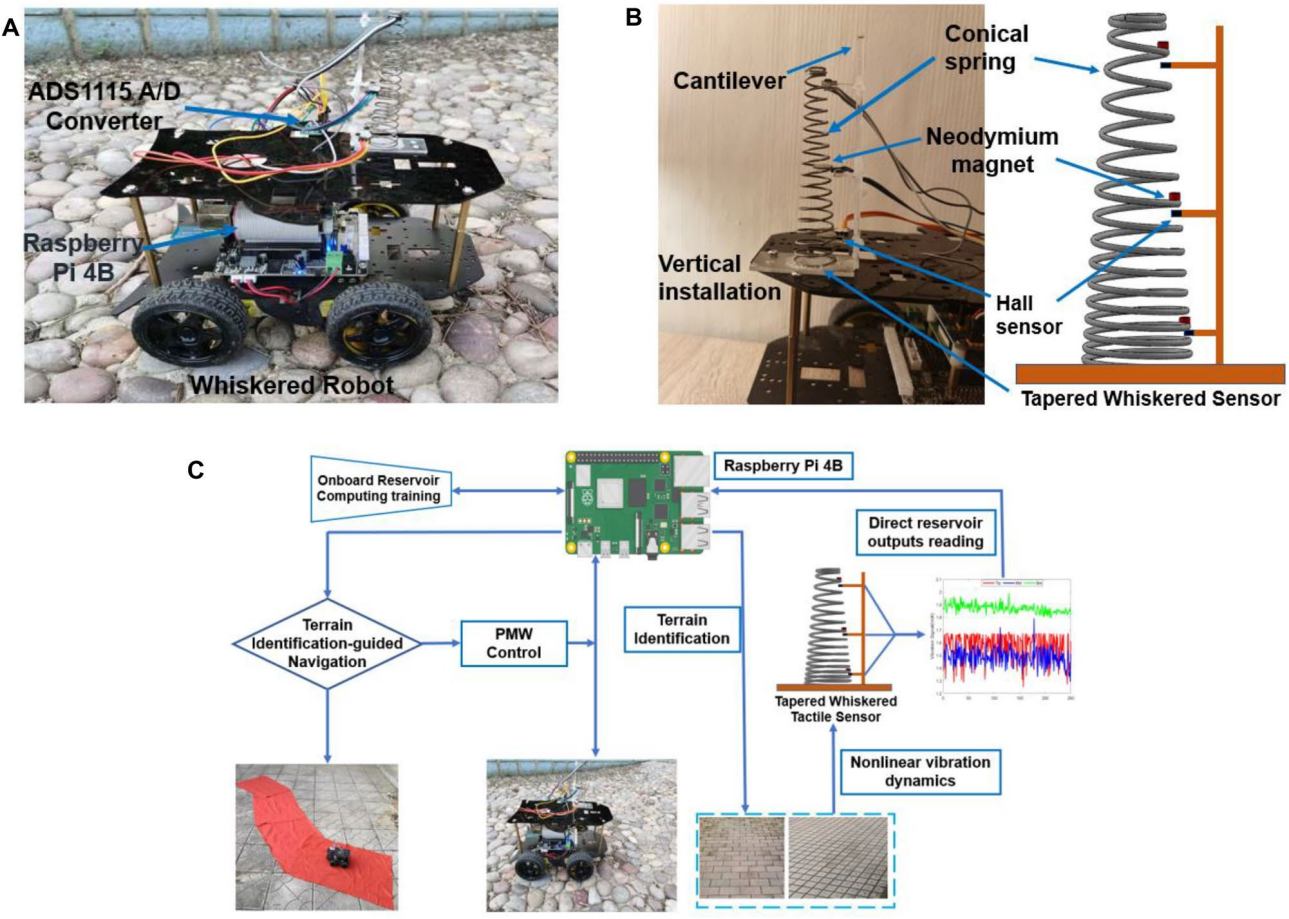
(**A**) A differential-drive mobile robot with a bio-inspired tapered whisker sensor vertically installed on the front to achieve reservoir computing-based terrain classification and texture-guided navigation. (**B**) The tapered whisker sensor and model. (**C**) Workflow of the proposed tapered whisker-based reservoir computing systems for the whiskered robot real-time terrain classification, and the terrain identification-based navigation.

Taking inspiration from nature another approach has recently emerged called physical reservoir computing methods^31,32^. Physical reservoir computing exploits the nonlinear dynamics of a physical system to emulate a given nonlinear dynamic function. As depicted in Fig. 1A, at the centre of a physical reservoir computing system is a high-dimensional complex physical system (aka the *reservoir*), which takes an input (e.g., time-varying forces) and maps it nonlinearly into its high-dimensional state space^33^. In addition, by being a dynamic system, it integrates the information available in the input signal. The remarkable feature of this setup is, that it is sufficient to add a simple linear static readout (weighted sum) from the high-dimensional state space in order to achieve a desired computation (e.g., desired dynamical mapping). This means the complex task of learning to emulate a desired nonlinear and dynamic input-output mapping can be, with the help of a reservoir, reduced to simple linear regression^34^. Interestingly, a wide range of physical systems has been identified to be useful as reservoirs. This includes compliant body parts of robots^35^, networks of memristors^36^, and many others^37,38^. This means, physical bodies can be exploited and in a physical reservoir computing setup, the learning only consists of finding a set of linear output weights. This reduces significantly the required computational and can therefore be very useful for autonomous robots. In this paper, therefore, we use a nonlinear tapered spring as a physical reservoir. In our case the input is the force introduced by moving the robot over different terrains and therefore exciting the spring which is attached to it. For the readout we use three Hall sensors at different locations of the spring, see Fig. 1B, using the eigen feature matrix of the Hall sensors’ readout working as the input of the logistic regression training. By exploiting the nonlinear dynamics of the tapered whisker, we are able to learn to classify different terrain relying solely on simple, linear regression.

**Figure 3:**
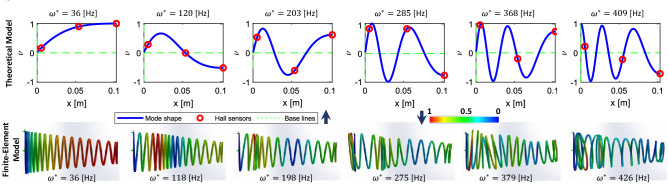
System modal response for the first six axial modes of vibration, with $\nu$ is the spring axial displacement and natural frequency $\omega ^*$, along the major axis based on the solutions from top) the theoretical framework, and bottom) FEA using the SolidWorks Static Analysis plugin. Please note that the top plots do not represent the spring’s deformed shape but the local axial displacement along the spring’s major axis.

**Figure 4:**
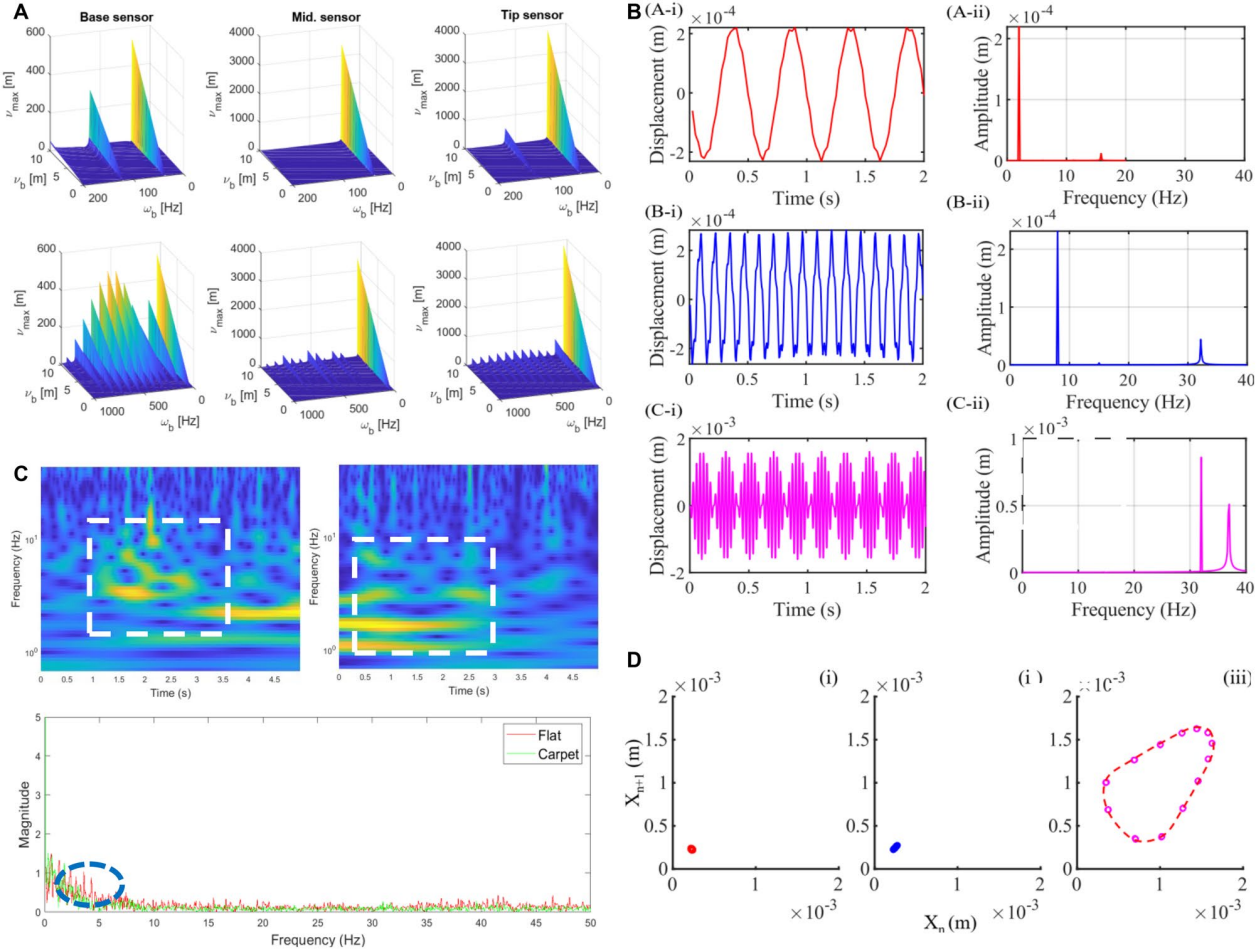
Theoretical study and quantitative experiments simultaneously verified the effect of dynamic response at different whisker axis locations on signal separation. (**A**) Parametric study of the maximum beam lateral displacement $\nu _{max}$ at the location of the three hall sensors (at the spring base, middle, and tip) for various base sinusoidal excitation signal amplitude $\nu _b$ and frequency $\omega _b$. Top) low-frequency range ($\omega _b < 200$ Hz) plots to highlight the excitation due to the train texture. Bottom) higher frequency range plots associated with texture/sensor noise and mobile robot structure vibrations. (**B**) Displacement waveform (i) and power spectral density (ii) plots; (A) $f=2\,{{\rm Hz}}$, (B) $f=8\,{{\rm Hz}}$, (C) $f=32\,{{\rm Hz}}$. (**C**) The top two sub-figures are the continuous wavelet transform (CWT) analysis of the top Hall sensor’s outputs of the whisker sensor as a result of the motion of the mobile robot on flat and carpet terrains respectively. The bottom figure is the FFT analysis results of the vibration signals over these two terrains. The dominant vibration modes have a frequency of $\omega _b < 20$ Hz. (**D**) The return maps of the system dynamics corresponding to base excitations; (i) $f=2\,{{\rm Hz}}$, (ii) $f=8\,{{\rm Hz}}$, (iii) $f=32\,{{\rm Hz}}$. The return maps show a plot between the first local maxima ($X_n$) of the displacement waveform to the next local maxima ($X_{n+1}$) of the displacement.

**Figure 5:**
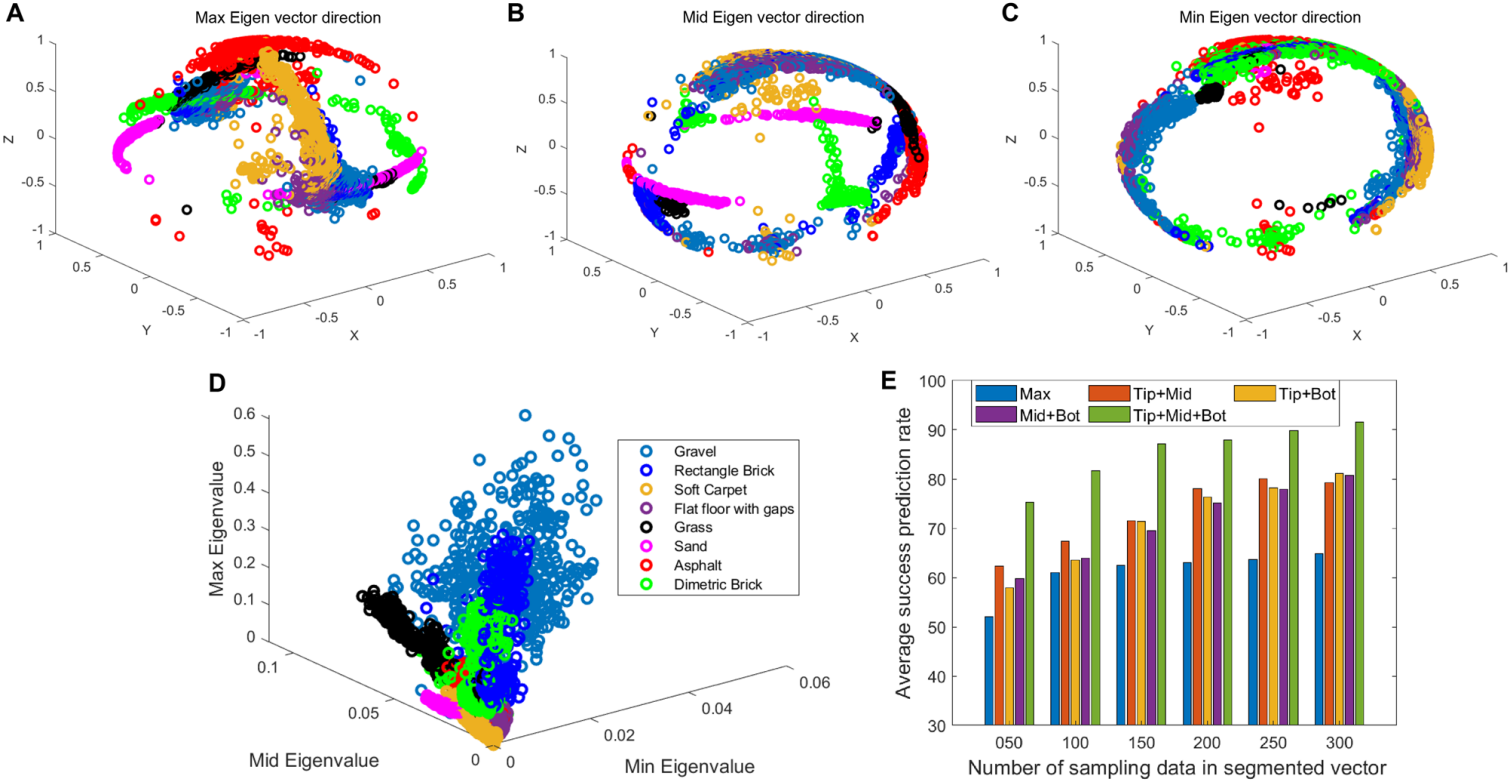
(**A**) The maximum-eigenvector direction of the whisker reservoir outputs. (**B**) The medium-eigenvector direction of the whisker reservoir outputs. (**C**) The minimum-eigenvector direction of the whisker reservoir outputs. (**D**) Eigenvalue of the TWRC system of eight different terrain surfaces in Eigenspace. (**E**) The average RC system prediction success rate of the different paired Hall sensor reservoirs of different time windows *T* based on its Eigenvector, and the results based on the maximum-eigenvector of three Hall sensors.

To our knowledge, this is the first time that a reservoir computing-based navigation system employing a tapered whisker as the reservoir computer is deployed in a mobile robot for fast real-time terrain identification and practical texture-guided navigation tasks relying solely on an onboard Raspberry Pi 4B single board computer rather than an external computer (Fig. 2), which is the main contribution and significant advancement compared with our previous work^39,40^. Another important improvement is that we quantitatively and qualitatively evaluate the physical reservoir computing capabilities of the proposed system, especially the frequency separation analysis in various analytical models and Finite Element Analysis (FEA) frameworks (Figs. 3, 4). We quantitatively demonstrate the accuracy of our proposed model analysis through experiments, while experimentally exploring the effect of dynamic response outputs for different whisker axis locations and their different output combinations on the classification results in Figs. 4C and 5. We demonstrate that our proposed algorithm could cost-efficiently achieve highly accurate real-time terrain classification results in Fig. 5B compared with state-of-art methods, and analyzed and demonstrated experimentally how the mobile robot can be controlled by speed to elicit unique frequency domain responses in a whisker sensor to help surface identification in Fig. 5C. Finally, as illustrated in Fig. 7, we demonstrate that the reservoir computing system could provide morphological computation power for real-time surface texture-guided mobile robot navigation solely relying on onboard hardware rather than an external computer.

## Results

### System modal response and parametric study

The external excitation of a nonlinear spring reservoir should replicate a reservoir computer composed of a large number of random and fixed internal nonlinear nodes. As a result, the computing system only needs to be trained to estimate the output signal weight matrix $W_{{\textrm{out}}}$ to relate the reservoir states *x*(*t*) and the output signal *y*(*t*). In this section, the dynamic response of the proposed tapered whisker is investigated in various theoretical and Finite Element Analysis (FEA) frameworks to comment on the physical reservoir computing capabilities of the proposed system. The results are compared with those of a simple spring-based whisker used in our previous study^17^.

#### Steady state modal response and sensor placement

The system modal response for the first six natural frequencies is plotted in Fig. 3. These values and plots are compared against the results from a modal FEA of the 3D system using the SolidWorks static simulation plugin. Here, only the modes with dominant axial deflection are plotted. The values for $\omega ^*$ and the overall mode shapes are comparably close between the theoretical and the FEA results with 7.5 Hz mean absolute (2.5% w.r.t the FEA results) error. This suggests that the provided theoretical framework is adequate for further structural dynamic analysis of the actual system.

A Fast Fourier Transform (FFT) analysis of the experimental results showed that the dominant vibration modes from various types of ground surfaces have a frequency of $\omega _b < 20$ Hz. Higher modes are associated with disturbances and sensor noises with negligible amplitude. This suggests that the first two mode shapes contribute the most to the response of the system. Hence, three sensors along the spring are adequate to gather enough information for decoding the system excitation source. Next, the theoretical framework presented is utilized to highlight the reservoir computing capabilities of the system in parametric studies which are very time-consuming to perform otherwise due to the high computational cost of FEA.

#### Excitation parametric study on sensor signal

The sensors’ signals are proportional to the maximum beam displacement at the axial location of the sensors. To investigate the relationship between the Hall sensors’ response to different beam base excitation, the maximum displacement of the beam at the sensor locations $l_H$ (6.5 mm, 55 mm and 103 mm from the base) are plotted against different values for the base excitation amplitude $\nu _b$ and frequency $\omega$ in Fig. 4A. The beam steady-state response based on the presented theoretical framework is used in this parametric study.

The low-frequency range plots ($\omega < 200$ Hz, Fig. 4A-top) show that the base sensor signal follows the excitation signal, as expected due to the location of the sensor. The middle sensor is located close to a node of the second mode shape while the tip sensor is never on a mode shape node. This results in a significant difference in the signal amplitudes (and hence the information richness) of the set of sensors. We suspect that these differences in the sensor signals provide enough nonlinearity required for a reservoir computer and enough information for the train identification task.

The high-frequency range plots (Fig. 4A-bottom) show a small amplification factor for the middle and tip sensor locations (hence, signals) compared to the low-frequency range plots and the base sensor readings. This highlights the desirable low-pass filter, i.e. amplification for train texture relevant excitation and filtering for higher frequency noises, properties of a vertical tapered helical spring structure compared to a vibration sensor attached close to or directly on the excitation base, e.g. the mobile robot chassis in this case. This suggests that the sensors should be robust enough against (by not amplifying) low amplitude but high-frequency excitation signals with $\omega _b < 2000$ Hz, that may occur due to the train imperfections and mobile robot structure vibrations.

The analysis results of the actual terrains experimental tests in Fig. 4C demonstrate the accuracy of our previous analysis of the dynamic response of the tapered whisker sensor. An FEA study for the dynamic response of the system is conducted in the next subsection to investigate the transient dynamics of the system. This is important to evaluate the robustness of the system’s response to sudden disturbances.

#### Transient dynamics finite element analysis

A vibration analysis of the whisker was performed using SolidWorks software. Initially, the whisker was modelled using SolidWorks and imported into SolidWorks Simulation. Thereafter, the model was discretized and a mesh was generated. A base exciting acceleration *a*(*t*) was applied to the base of the conical spring. The acceleration *a*(*t*) is given by $a(t) = sin(2 \pi f t)$, where *f* is the frequency of vibration and *t* is time. Then, a non-linear transient simulation with a varying time step was undertaken. The frequency of vibration varied from 2 Hz to 64 Hz. To comply with the actual whisker properties, spring stainless steel (AISI 304) was used as the material for the vibration analysis.

Figure 4B shows the displacement waveform and power spectral density plots of the topmost whisker position for frequencies of 2 Hz, 8 Hz, and 32 Hz. As the forcing frequency increases from 2 Hz to 32 Hz, the motion transforms from periodic oscillations to quasiperiodic oscillations as shown in waveform plots. This arises due to the natural frequency of oscillations becoming dominant as shown in power spectral density plots. When the base exciting frequency increases, the amplitude of the second-dominant frequency increases. This results in a quasiperiodic oscillation of two dominant frequencies which could be observed in Fig. 4B,C. Figure 4D shows the return maps (Poincaré map) for frequencies of 2 Hz, 8 Hz and 32 Hz which map the first local maxima of displacement waveform to the next local maxima. This gives insight into the displacement properties of the whisker by lowering its phase space dimension. The first two return maps show sets of dense localized points which correspond to periodic oscillations. The third return map shows a closed loop structure which corresponds to a quasiperiodic oscillation. This suggests that the underlying dynamics of the whisker under base excitations are not chaotic.

### Experiments analysis of tapered whisker dynamic response

In order to verify the aforementioned theoretical and FEA analyses on the physical reservoir computing capabilities of the proposed system, we conducted a variety of terrain classification experiments to explore the effect of dynamic response outputs for different whisker axis locations and their different output combinations on the classification results. In addition, we also explored the depth of the effect of movement velocity on frequency separation of the whisker reservoir computing system and its impact on terrain classification accuracy.

#### Dynamical response of different whisker axis location

By comparing the maximum, medium and minimum eigenvector direction of the whisker reservoir outputs shown in Fig. 5A,B,C respectively, we can find that the maximum eigenvector direction can distinguish eight different terrains significantly, while the minimum eigenvector would have a lot of chaos and signal overlap, which will cause misunderstandings for classification. This result shows that the direction of the sensor output eigenvectors can provide different surface texture information, and different surface information can produce completely different eigenvector directions. Fig. 5D gives the eigenvalue of our proposed TWRC system when it traversed eight different terrains. This indicated that varying external surface stimuli from different roughness and hardness terrains induce distinct eigenvalues for our proposed tapered whisker TWRC system. All these results also mean the whiskered mobile robots could autonomously identify and vaguely predict the roughness and softness of the terrain it traversing, only based on the prior calibrated tapered spring whisker RC system.

These results reinforce our previous theoretical analysis that the tapered whisker could provide enough nonlinearity required for a reservoir computer and enough information for the train identification task. This might also indicate that the nonlinear stiffness of the tapered whisker can be controlled to rotate the covariance direction between different pair Hall sensors for a certain external vibration to improve the classification accuracy. To demonstrate this, we employed different paired Hall sensor outputs to train the logistic regression network and compare the classification results. The results in Fig. 5E show the average TWRC system prediction success rate of the different paired Hall sensor reservoirs of different time windows *T* based on its eigenvector, and the results based on the maximum-eigenvector of three Hall sensors. Terrain classification results will vary for different paired Hall sensor reservoirs for the same time window length, which implies that the non-linear stiffness of the tapered whisker sensor is an important internal natural parameter and that combinations of the outputs of different stiffness positions can elicit information perception in our mobile robot. Fusing different paired stiffness reservoirs- outputs would help the robot to capture different frequency information in the time domain directly to better perform surface classification tasks.

**Figure 6:**
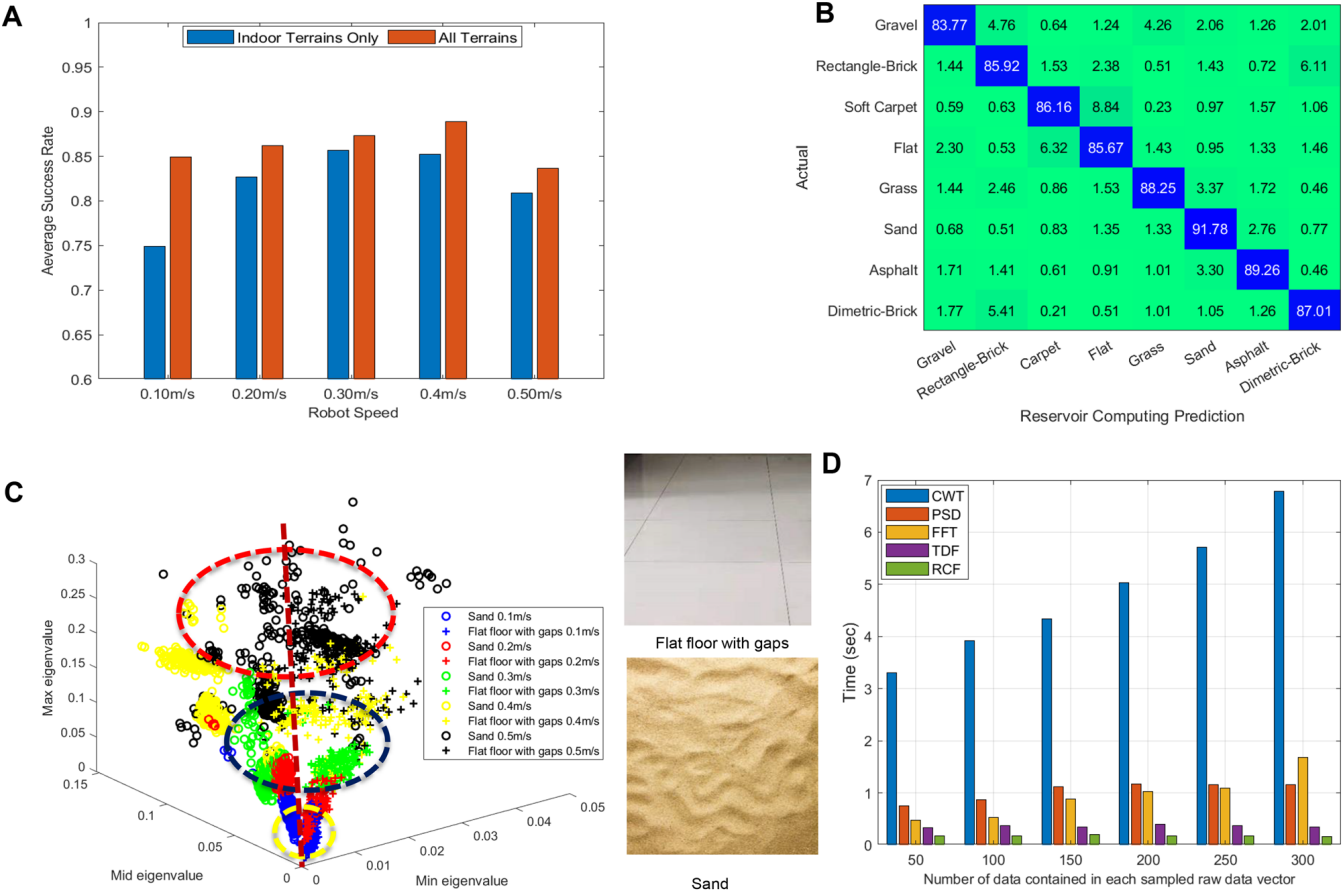
(**A**) Reservoir computing average prediction accuracy of eight terrains at varying robot speeds: 0.1 m/s, 0.2 m/s, 0.3 m/s, 0.4 m/s, and 0.5 m/s. (**B**) Average classification results of the TWRC system over 25 random trials when the robot moving at 0.2 m/s and the time window is 1.5 s. (**C**) The effect of speed on the separation of Eigenvalue vectors for soft sand and flat floor with gaps. (**D**) Experimental process time comparison of different feature extraction methods for processing 1000 sampled raw data vectors with different time window lengths *T*. PSD means power spectrum periodogram, TDF means time domain features, and RCF represents the reservoir features.

#### Effect of robot speed on signal separation

As shown in Fig. 6A, we conducted experiments over eight different terrains with several stable speeds of 0.1 m/s, 0.2 m/s, 0.3 m/s, 0.4 m/s and 0.5 m/s respectively to explore the effect of speed on the classification accuracy. If the speed is too large (i.e. more than 0.4 m/s) or too small (i.e. less than 0.2 m/s) the classification accuracy drops. This shows that speed of movement is a control variable to optimally apportion vibration frequency components across the 3 sensors placed along the tapered spring.

Figure 6B gave the classification confusion matrix of our TWRC system for 25 random experiments, with the average classification rate of $87.2\%$ for the time window 1.5 seconds. These results demonstrate that the TWRC system has effective identification and classification capabilities even for very similar terrain surfaces, which means that it can be applied to a wider range of scenarios to execute robot deployment tasks, such as object classification and texture information capture. Compared with the results in Fig. 5E, this reports a significant improvement in classifications of these eight terrain surfaces, which reflects the fact that both the eigenvalues and the eigenvectors contain texture information that can reflect external stimuli.

As shown in Fig. 6C marked by a yellow circle, when the speed is relatively low, the different external terrain profiles result in relatively small and overlapping tapered whisker reservoir features, making it difficult to distinguish accurately, and the vibration generated by the robot itself will have a relatively large disturbing effect on it at this time. This is because when the whisker sensor is in this stiffness state and the operating speed is too low, the whisker vibration caused by the change of the terrain surface profile will be relatively weak and smooth. Moreover, in our experiments, when the speed reaches 0.5 m/s, its classification accuracy is significantly lower relative to that at 0.4 m/s. This may be due to the fact that when the whiskered robot speed is too fast, the vibrations generated in the whisker sensor due to different terrain surfaces may result in a chaos of reservoir features as marked by a red circle in Fig. 6C, which is essentially chaos in the frequency domain, leading to difficulties in accurate differentiation. Combining the results shown in Fig. 6A,C indicates that a mobile robot could use speed control to elicit unique frequency domain responses in a tapered whisker sensor to help surface identification.

#### Computational superiority of the proposed reservoir system

To demonstrate the computational efficiency of our reservoir computing (RC) approach with the state-of-art feature extraction methods IMU accelerometer^12^, microphone audio^14^, or tactile probe-vibration data^15^, we compared the time required by these above algorithms to process the same amount of raw data in Fig. 6D. The computation results in Fig. 6D show that to extract the same number of feature matrices, using the RC method is much faster and more computationally efficient, compared to the aforementioned traditional methods CWT, FFT, PSD, and TDF (we extract four time features of raw data in this experiment: mean, variance, root mean square, integral of absolute value). Even if the TDF extracting features in the time domain is the fastest compared with the aforementioned traditional methods CWT, FFT, and PSD, our RC feature extraction method is at least $60\%$ faster in actual computation compared with the TDF. Moreover, these methods not only require computation resources to process the sampled data and extract surface features, but a complex deep learning network is also still needed for model training for most state-of-art methods to perform surface identification and classification. In contrast, the output of RC can be directly used for simple linear regression to obtain fast classification and recognition results with cost-efficient computation sources, as illustrated in Fig. 2, which is a computational economy approach based on theoretical computational complexity compared with other neural networks like convolutional neural Networks, deep neural network. These experiments are finished by using Matlab 2020a on a computer (Intel(R) i5-10210U CPU@1.60 GHz, 8 GB RAM), and 10 trials have been performed for each extraction method to ignore the influence of the operating compute system load.

### Whisker reservoir computing based navigation experiments

**Figure 7:**
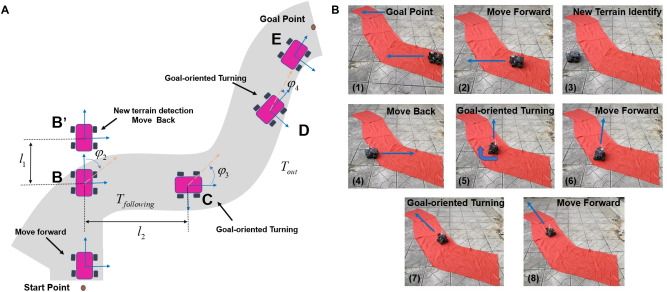
(**A**) Surface texture-guided navigation algorithm to follow a terrain surface $T_{following}$ (light grey) based on the real-time TWRC system. (**B**) Time series video images taken during terrain texture identification-guided mobile robot navigation experiment, following the red carpet surface. These images, from (1) to (8), demonstrate one of the successful texture-guided navigation manoeuvres. In (3), the whiskered robot detected the surrounding terrain $T_{out}$ and executed the *Goal_oriented_Turning* operation in the image (5). In (7), the robot performed a final steering manoeuvre to reach its final destination in (8).

To validate that the tapered whisker-based reservoir computing system can be employed for fast real-time terrain identification and practical texture-guided navigation tasks relying solely on the onboard Raspberry Pi rather than an external computer, we proposed a whisker reservoir computing based navigation strategy shown in Fig. 7A and conducted a surface texture-guided navigation experiments in Fig. 7B. In this experiment, a red carpet 1 m wide and 3 mm thick was laid in an *S*-shape on top of the brick terrain to serve as a following terrain $T_{following}$, as shown in Fig. 7B. We used the same configuration differentially driven mobile robot equipped with the TWRC system as in the previous experiments to perform this surface texture-guided navigation experiment by using the *Navigation algorithms*. Figure 7A gives the detailed navigation strategy. A new reservoir computing weight matrix was retrained by adding the new red carpet class for fast terrain recognition to avoid driving off the terrain $T_{following}$ , and the control commands for the mobile robot will be sent directly to the wheel servos via the Raspberry Pi. In order to avoid the impact on terrain recognition caused by the robot’s vibration during stopping and steering, the whiskered robot is stopped for a period of time after each manoeuvre to allow the tapered whisker sensor to regain stability.

As can be seen in Fig. 7B, the whiskered robot can successfully reach the target point by following the specified complex-shaped terrain with high quality. By performing multiple *Goal_oriented_Turning* turns, the mobile robot can avoid traversing the surface of the brick based on the rapid recognition of the anomalous terrain by the TWRC algorithm. The success of this experiment relies on our reservoir computing algorithm based on the tapered whisker sensor to identify the terrain with high accuracy and rapidity, and our *Navigation* algorithm to precisely control the robot’s movements. In order to test the reliability of our algorithm, we conducted repeated experiments in the scenario shown in Fig. 7B. Although the robot path of each experiment robot will be different due to the influence of the starting point location and the drift error due to accumulation over time, the mobile robot can quickly recognize unknown terrain and keep moving on special terrain $T_{following}$. These experiments’ results indicate that robots can achieve independent and autonomous motion planning and obstacle avoidance without relying on any external high-performance computing devices by using only a simple reservoir computing system on a Raspberry Pi 4B single-board computer, which is of great importance for robots performing tasks in extreme environments with limited computing resources, such as, for example, Mars exploration. More sophisticated and accurate navigation algorithms can be developed in our future research, thus allowing our TWRC algorithm to be applied to more scenarios.

## Conclusion

In this paper, we presented a real-time terrain identification-based navigation method for mobile robots using an onboard tapered whisker-based reservoir computing system as the core, relying solely on an onboard Raspberry Pi 4B single-board computer rather than an external computer. Terrain surface following experiments demonstrated that our method is fast and accurate enough to identify changes in terrain and adjust their trajectory in time to follow specific shapes of the terrain. This is a new terrain recognition-based navigation technology compared to alternative terrain recognition-based navigation technologies (e.g. vision).

Through theoretical analysis, based on the Euler-Bernoulli beam theory, and Finite Element simulations, we have demonstrated that a nonlinear conical whisker sensor can directly implement a reservoir computer for frequency separation from time domain vibrations in the mobile robot. Through experiments, we explored the dynamic output of different whisker axis locations and their different combinations, as well as the effect of different motion speeds on the separation of external signals, especially for terrain classification accuracy. Experiments also proved the effectiveness and the computational superiority of the method, and the robot could adapt to the optimal movement speed depending on the scenario.

Future work involves improving the design of the whisker sensors to improve the classification accuracy of the terrain. One interesting direction is to design high-performance whisker sensors by optimizing the locations of tapping the whisker sensor signal outputs. Another approach is to investigate the use of real-time stiffness control of the whiskers as a control parameter to excite the steady-state vibration frequency components specific to a given terrain. In addition, we can improve the robot’s ability to perform tasks in different scenarios by fusing signals with other sensors, such as vision and audio.

## Methods

### Tapered spring-based whisker design

The proposed nonlinear tapered spring-based whiskered sensor for reservoir computing in this paper is illustrated in Fig. 2B. The whisker sensor was constructed by a high-carbon (S. Steel AISI 304) tapered nonlinear spring with the wire diameter *d* is 1 mm, which also has the free length is *l* 100 mm and the spring number of coils *n* is 18, and the coil base mean radius $R_b$ and tip mean diameter $R_t$ are 20 mm and 10 mm respectively. The beam bending modulus *EI* is equal to the spring wire torsional modulus *GJ*, in which $G=E/3=70$ [GPa] is the wire material (stainless steel) shear modulus, and the $J=\pi d^4 / 4$ is the wire cross-section 2nd moment of area. The wiring density $\rho$ is $8050\,\textrm{kg}/\textrm{m}^3$ and the moment of area correction factor $c_J$ is 0.08. To accurately capture the external oscillation information, three linear Hall sensors (SS49E) are orthogonally installed under the three permanent neodymium magnets at the locations $l_H$ (6.5 mm, 55 mm and 103 mm from the base), which were embedded in the top, middle, and bottom of the tapered whisker to capture continuous magnetic field changes nearby when there is external excitation, as shown in Fig. 2A,B. The tapered whisker sensor is orthogonally mounted in the left front of the mobile robot, as shown in Fig. 2A. The external vibration caused by robots steadily traversing different terrains will work perpendicularly on the tapered whisker shaft, which will lead to nonlinear vertical displacement of the magnets along the spring beam, as shown in Fig. 1C. Figure 1B demonstrates the computation framework of the physical tapered whisker reservoir computer system, in which the Hall sensor will capture the magnetic flux change induced by the nonlinear vibration to provide morphological computation power.

### Experiment platform and procedure

The overall system diagram of the tapered whisker reservoir computing for real-time terrain identification-based navigation is shown in Fig. 2C. The tapered whisker-based reservoir computing, terrain identification and robot navigation and control are synchronized and conducted online by the Raspberry Pi 4B, because the reservoir computing system only requires modest computing resources for linear readout weight training, the mobile robot could synchronously adjust its trajectory based on the real-time terrain identification result to avoid falling into dangerous terrains. The detailed navigation algorithm will be presented in Fig. 7A, and the details of the data collection, processing, and weight metrics training could be found in the subsection of data collection and RC training. In this paper we use the same robot configuration for all experiments. For all the experiments, the whiskered robot moved over the eight different terrains at a steady speed with the sampling frequency is 100 Hz to collect training datasets for terrain surface classification and navigation, as illustrated in Fig. 2.

### Theoretical analysis of whisker dynamic response

Parametric studies for the problem of the dynamic response of a tapered whisker sensor to a harmonic base excitation cannot be performed in a reasonable time based on high-fidelity FEA. In this section, a simple analytical framework is presented to investigate the steady-state solution to this problem (see Fig. 1C).

#### Axial base excitation of a tapered cantilever beam

In this section, an analytical model is presented for the axial vibration of a tapered spring following the derivations in^41^. The equation of force equilibrium, between the inertial and spring tension forces, for an element of this system not subject to external force, takes on the form

1 $$\begin{aligned} \frac{2 \pi m Z^4}{a^2 G d^4} \frac{\partial ^2 \nu }{\partial t^2} = \frac{\partial ^2 \nu }{\partial Z^2} - \frac{3}{Z}\frac{\partial \nu }{\partial Z}, \end{aligned}$$

where $\nu$ is the axial displacement of the element, $Z = 2(R_b-\alpha ) x$, $\alpha = ( R_b - R_t ) / l$ is the tapered slope, $R_b$ and $R_t$ are the spring base and tip mean radius, *l* is the spring length, $m = \rho a$ is the wire mass per length, $\rho$ is the wire density, $a = \pi d^2/4$ is the wire cross section area, *d* is the wire diameter, and *G* is the wire material shear modulus.

Following the separation of the variable method, a general solution based on the first-order cylindrical (Bessel) functions *J* of orders 2/3 and $-2/3$ are derived in^41^ as

2 $$\begin{aligned} \nu (z,t) = (\frac{\Lambda }{2})^{\frac{2}{3}} \zeta ^2 \left( \mathscr {C}_1 J_{-\frac{2}{3}}(\Lambda \zeta ^3) + \mathscr {C}_2 J_{\frac{2}{3}}(\Lambda \zeta ^3) \right) \cos (\lambda t + \delta ), \end{aligned}$$

where $\zeta =Z/(2R_b)$, $\Lambda = \lambda /(3 \sqrt{c})$, $\lambda =2 \pi \omega$, $\omega$ is the harmonic response frequency in Hz, $c=\alpha ^2 G d^4 / ( 128 \pi m R_b^6 )$ is a bulk constant, $\delta$ is the harmonic response phase difference, and $\mathscr {C}_i$ is a constant.

This solution can be extended to the case of the steady-state response of the base axial excitation of a tapered spring, which is the target of this analysis, as long as the base excitation and the beam harmonic response have the same frequency $\lambda$. Then the base excitation of the form $\nu _b \cos (\lambda t + \delta )$ can be enforced as a boundary condition for the axial motion of the spring base. Then, the boundary conditions at the spring base and tip are

3 $$\begin{aligned} \nu (0) = \nu _b,\ \partial \nu / \partial x = 0, \end{aligned}$$

where $\nu _b$ is the amplitude for the base axial vibration and the second boundary condition stands for the load-free condition at the spring tip.

Substituting Eqs. (3) in (2) results in a system of equations as follows

4 $$\begin{aligned} \mathscr {A} \cdot \begin{bmatrix} \mathscr {C}_1&\mathscr {C}_2 \end{bmatrix}^\top = \begin{bmatrix} \nu _h/(\frac{\Lambda }{2}^{\frac{2}{3}}\zeta _b^2)&0 \end{bmatrix}^\top , \end{aligned}$$

where $$\mathscr {A}$$ is

5 $$\begin{aligned} \begin{bmatrix} J_{-\frac{2}{3}}(\Lambda \zeta _b^3) &{} J_{\frac{2}{3}}(\Lambda \zeta _b^3) \\ 3 \zeta _t \Lambda \zeta _t^2 J_{-\frac{2}{3}}'(\Lambda \zeta _t^3) + 2 J_{-\frac{2}{3}}(\Lambda \zeta _t^3) &{} 3 \zeta _t \Lambda \zeta _t^2 J_{\frac{2}{3}}'(\Lambda \zeta _t^3) + 2 J_{\frac{2}{3}}(\Lambda \zeta _t^3) \\ \end{bmatrix}, \end{aligned}$$

$\zeta _b=\zeta (0)$, $\zeta _t=\zeta (l)$, and from the recursive property of Bessel functions $J_v'(x) = \partial J_v(x) / \partial x = \left( J_{v-1}(x) - J_{v+1}(x) \right) / 2$.

#### Axial displacement and natural frequencies

In the case of base axial excitation, we may solve the inverse problem form Eq. 4 for the values for $\mathscr {C}_i$ given the known base axial excitation frequency $\omega$ (hence $\Lambda _b$) and amplitude $\nu _b$. The system natural frequencies $\omega ^*$ can be found by solving for the frequencies that satisfy the following relation $\textrm{det}(\mathscr {A})=0$, where $\textrm{det}$ is the matrix determinant operator.

**Table 1:** Tapered spring whisker parameters.

Info	Symbol [unit]	Value
Wire radius	*r* [m]	5e–4
Spring length	*l* [m]	103e–3
Hall sensor locations	$l_H$ [m]	[6.5, 55, 103]e–3
Spring coils	*n*	18
Coil base mean radius	$R_h$ [m]	20e–3
Coil tip mean radius	$R_g$ [m]	10e–3
Wire shear modulus	*G* [Pa]	70e9
Density	$\rho$ [Kg/m$^3$]	8050
Moment of area correction factor	$c_J$	0.08
Wire material	–	S. Steel AISI 304

#### Equivalent parameters for a compression spring

The above derivations assume each full coil as a finite element along the spring axis. To accommodate the effect of the spring number of coils $n_c$ and pitch $p=l/n_c$, we may substitute the spring length *l* with $n_c$ in the above relations. Hence, we have $\alpha = (R_0 - R_1)/n_c$ and $x=x_c p$, where $x_c$ is the unit length based on the coil number. Table 1 presents the tapered spring whisker parameters used in our theoretical and FEM studies, which are based on the average reported values for Stainless Steel material in the literature.

### Data collection and logistic regression retraining

To verify that our robot relying on the TWRC system can follow a specific terrain surface and quickly recognize new terrain, we collected training data on the red carpet shown in Fig. 7B in order to train a new reservoir-computing- weight-matrix. The whiskered robot moved over the red soft carpet at a steady speed of 0.2*m*/*s* with the tapered whiskered tactile sensor mounted on the same place during experiments, the mobile robot configuration is the same as our previous experiments. We use the same ADS1115 A/D converter to connect the vibration data with the sampling frequency is 100 Hz. After that, the covariance matrix $C{_D}$ of collected sampling vector $D_k^{m\times 3}$ was then decomposed into their Eigen representation for logistic regression weights training.

6 $$\begin{aligned} C{_D} =cov_{p,q}= {R_{vec}}{R_{val}}R_{vec}^T \quad p,q = T,~B,~M, \end{aligned}$$

where the eigenvalue ${R_{val}}$ representing the scaling factor in each dimension of $D_k^{m\times 3}$, and the eigenvector ${R_{vec}}$ of the $C{_D}$ reflecting the dispersion direction of the distribution of the $D_k^{m\times 3}$. The reservoir feature matrix ${R_{f}}$ working as the input of the logistic regression is given as follows:

7 $$\begin{aligned} {R_f} = [{R_{vec}} \quad {R_{val}}]^{T} \end{aligned}$$

Only the simple logistic regression network was employed to train the readout weights $W_{{\textrm{out}}}$ for the different terrain recognition, as shown in Fig. 1. A, and the highest probability of the expected possible terrain surfaces would be cost-efficient determined both by the input reservoir vector ${R_{f}}$ and the weight vector $W^*_{{\textrm{out}}}$. To avoid inaccurate recognition due to data imbalance, we used the same amount of data as the other terrain categories to train the weight matrix for logistic regression, the time windows of each sampled terrain vector are 1.5 s with 150 vectors in total for each class. More details about the data processing and logistic regression training can be found in our previous paper^42^.

### Real-time terrain identification-based navigation strategy

We proposed a surface texture-guided navigation algorithm based on the TWRC system to follow a specific terrain surface. Fig. 7A gives the detailed navigation strategy, when the whiskered robot is on the specified terrain surface $T_{following}$, it will move towards the target point at a specific speed ${v_{robot}}$. Our on-board TWRC system can immediately identify the terrain currently being passed due to its not requiring complex data pre-processing prior to being used for classification. Moreover, the mobile robots could also accurately estimate the property of the unknown terrain surface and achieve auto-labelling of new terrain accurately estimate the property of the unknown terrain surface based on our previous work^40^. When the robot realizes that it is driving away from surface $T_{following}$, it will immediately stop and move back a distance $l_1$ in a straight line to return to the $T_{following}$.

When the whiskered robots move back to $T_{following}$, the *Goal_oriented_Turning* module will control the whiskered robot to rotate in place towards the target based on the rotation angle $\varphi$ relative to the target ${P_{goal}}$. The scalar and direction of the rotation angle $\varphi$ will be calculated based on the relative position of the current position ${P_{current}}$ to the target position ${P_{goal}}$. For example in Fig. 7A, at point **B** the ’whisker’ robot will do a clockwise rotation of angle $\varphi _2$, while at point **C** for point **D** it will do a counterclockwise rotation of angle $\varphi _3$ and $\varphi _4$ respectively. In order to avoid the impact on terrain recognition caused by robots vibration during stopping and steering, the whiskered robot is stopped for a period of time after each manoeuvre to allow the tapered whisker sensor to regain stability. The location of the whiskered robot ${P_{current}}$ will be incorporating calculated speed, heading direction, and course over elapsed time using the dead reckoning method. Considering the small size of our test site, the effect of drift error due to accumulation over time can be ignored. Based on this navigation algorithm, our whiskered robot can successfully reach the target point by cruising the specified terrain surface through several *Goal_oriented_Turning* attempts.

## Data Availability

The raw data and material used and analyzed in this study are available from the corresponding author upon reasonable request.
